# Questions Medico-Legal

**Published:** 1873

**Authors:** D. A. Morse

**Affiliations:** Midway, Ohio


### SELECTIONS.

### QUESTIONS MEDICO-LEGAL.

BY D. A. MORSE, M. D., MIDWAY, OHIO.

Does *antenuptial incontinence* and *venereal disease, existing at the time of marriage,* with one party, but unknown to the other, constitute a sufficient ground for divorce?

If one party enters into the marriage relation with another, not having been informed that the second party has, by an impure connection, contracted syphilis, will such marriage, in the light of a contract, be *void* or *voidable?*

Can the aforementioned conditions be brought, constructively or otherwise, within the meaning of the statutes which define the causes for which divorce shall be granted?

The law of Ohio, vol. 51, Stat. 377, passed March 11, 1853, declares, "That the several courts of common pleas in this State shall have the cognizance of granting divorces for the following causes: 1. Where either of the parties had a former wife or husband living at the time of solemnizing the second marriage; 2. Where either of the parties have been willfully absent from the other three years; 3. Adultery; 4. Impotency; 5. Extreme cruelty; 6. Fraudulent contracts; 7. Gross neglect of duty; 8. Habitual drunkenness for three years; 9. Where either party has been or shall hereafter be sentenced to imprisonment, and actually imprisoned in the penitentiary, etc."

Bouvier (Institutes, vol. 1. sec. 237) gives the following *conditions* or *qualities,* as those which render marriage *unlawful or voidable:*

1. Want of competent age; 2. Want of consent; 3. A former marriage existing; 4. Consanguinity or affinity; 5. Want of consent of parents; 6. Civil death; 7. Crime of adultery.

In a consideration of marriages that are voidable, these seem greatly deficient and imperfect; but if we examine the work further we find other causes given in the discussion of the above conditions, as in section 245, *incurable impotency* is declared a cause of divorce from "want of consent," etc.

As we shall have occasion to *illustrate the meaning* of the fifth and sixth *causes* given in the Ohio law, by the interpretation of a *similar* law in French courts, we give the parallel sections of the Code Napoleon, which does away with *divorce* but applies the same to what is termed a *separation of the bodies.*

"ART. 234. Les époux pourrout reciproquement demander le séparation pour excés, services et injuries graves de l'un d'eux envers l'autre."

The rule running through these cases, as applied by courts is that where certain causes are enumerated as grounds of divorce, that those concerning which the law is silent can not be alleged as sufficient grounds, the silence being construed as evidence that such causes are not deserving of legal consideration.

The questions propounded as the basis of this article unfortunately partake of this last feature; and unless we can show that the law implies, or *constructively* includes them, we must to each question give a negative answer.

How can we show unequivocally that the law may be construed so as to include these causes *not mentioned*, except the *general terms* used be understood to imply them, leaving it to the court to interpret what the terms "extreme cruelty" and fraudulent contract" mean?

First, then, to determine that the law *constructively* includes the causes in question, let us determine *what the law regards as marriage;* and, in the second place, ascertain if, *these conditions existing*, all the elements and ingredients of a lawful marriage are present.

*What is marriage?* Bouvier (Institutes, vol. 1, p. 101) says: "It is considered in this country as a civil contract simply, and not, as in some countries, a sacrament. As an institution established by nature, it consists in the free and voluntary consent of both parties, and in the reciprocal faith which they pledge to each other. As a civil contract, it not only requires that free consent shall be lawful, that is, conformable to the laws of the State where the contract takes place," etc.

Parsons on Contracts (vol. 2, p. 114) declares marriage to be a civil contract. Blackstone (Commentaries, vol. 1, sec. 433) says the law regards marriage only as a civil contract.

Chitty (Contracts, p. 468, 469) regards marriage as a contract, and says that a suit for breach of promise cannot be sustained if either party who failed to perform the contract discovered that the other was of bad character.

Addison (on Torts) says a marriage procured by false representations is a *tort*.

Bishop (Marriage and Divorce) takes a more rational view of marriage, and declares it to be *not a contract*, but a *status*. And in answer to the question, "What is marriage?" says, (sec. 3): "Marriage, therefore, so the author deems the term to be most truly defined, is the civil status of one man and one woman, united in law for life, under the obligation to discharge, to each other and the community, those duties which the community by its laws holds incumbent on persons whose association is founded on the distinction of sex."

Story (Conflict of laws, sec. 108) says: "Marriage is something more than a mere contract. It is rather to be deemed an institution of society, founded on the consent and contract of the parties; and in this view it has some peculiarities in its nature, character, operation, and extent of obligation, different from what belongs to ordinary contracts." This we regard to be the true view of marriage; for in what other relation in life have two parties no power to form or disavow a contract except by consent of the State, and yet the contract be *a simple civil agreement*. In this the sovereign will of the State is the governing power. Everything, although the contracting parties must yield *free consent*, must conform to this will, or all is void. Marriage, then, has a manifold relation to be considered: 1. That of the parties to each other; 2. To the offspring; 3. To society; 4. To the State; 5. To moral law.

Each of these relations must be preserved, or some ingredient of marriage is wanting. The law is founded upon the proper assemblage in marriage of these elements; thus an idiot, or one demented, can not *consent*. An incestuous union is void; it is a wrong to the parties, an evil in the moral world, and a corruption of social elements.

The end of marriage being the procreation and perpetuation of the race, all must centre in this and be subservient to it. As individuals compose the State, and as each individual enters life without his consent to form one of this great civil body, it becomes apparent that the will of the State should govern the relation of those who thrust their offspring upon its mercies, and claim its protection.

Can it justly, then, be considered a *contract* between two parties when a third party must be consulted, whose interests must be preserved, whose reputation must be unsullied, whose very *existence* depends alone upon its due observation and performance.

The very *existence* of the State seem to underlie the marriage relation; and the preservation of this marriage relation is the only guaranty of the preservation of its integrity as a body, and is the only safeguard of its liberties and moral power. It is from this principle emanates those laws dictating the basis of marriage and declaring the obstacles that forbid it. The State becomes the guardian of its own interests, that the greatest good, highest degree of happiness, peace and prosperity, may result to all. How is this interest in the marriage relation shown? Why has the State an unquestionable right to dictate the terms of marriage within her borders?

The *family* is the first *school* of the citizen, as well as the source from whence are derived each successive generation, who in their turn are to educate and prepare for the duties of citizens those who follow. The physical and mental integrity, as well as moral, of the *offspring*, depend upon the same conditions with each of the parents who bring them into the world: they are simple copies of their progenitors.

Again, *procreation* must sustain the stream that enters life to supply the never-failing tide departing. In this the State has the right to dictate that idiots, insane, diseased and helpless may not be her *burden* instead of her power and support. Hence, there must be a certain degree of physical, mental and moral perfection, lest the State diminish in numerical force, in intellectual vigor or moral strength. The State must, then, as a consequence, guard against such unions as will defeat her purposes, and endanger her safety or her interests. The result is, that the interests of the family and State are reciprocal. What are the conditions to be inferred from the above as proper to be declared as the basis of a legitimate union, and to which all others are *secondary* considerations? Simply what the common law has recognized—*unimpaired procreative ability.* Power to bring forth offspring—the absence of which renders a union voidable—is the *tie* that *unites the family*, and *preserves the interest of the State.* On the contrary, whatever impairs generative power, either by the production of impotence or sterility, or by rendering the production of viable germs impossible, should be an obstacle to marriage.

*Syphilis* is a disease that, while it does not render procreative power absent, does render it impossible to a great extent to bring forth healthy offspring. It destroys the health, if not the life of its victim; is a worse scourge to the innocent than a majority of the ills mankind are subject to. But venereal disease is not declared in the Ohio laws a cause for divorce. It is not mentioned in the Code Napoleon we have cited.

Although French law has no bearing on American cases, it seems eminently proper that when the language of such law is the same as an American law, has been in force for a long period, been subjected to every form of criticism, and has been interpreted by the higher courts, this interpretation and application in practice is worthy of consideration.

*What has been the French practice?* The relation of venereal disease to marriage was first considered in the French Parliament at Paris, in 1663. Whether syphilis should be regarded a cause for divorce was not decided. In 1757 the question arose in an inferior court, and it was declared that the disease in question was a sufficient cause of divorce. An appeal was taken to the Parliament at Paris, and on December 16, 1771, the judgment of the court was confirmed.

The ground upon which the case rested was that the case was brought within the meaning of the law which allowed a separation for "*excés, sevices et injuries graves*" (abuse, ill-treatment, grave injury), regarding syphilis as unequivocal evidence of libertinage, and if contracted *subsequent* to marriage, infidelity. The counsel said, "What cause more legitimate for separation than the danger threatened to the healthy party, and for the offspring, if any are born."

Fodere tells us that during the time of the Empire, under the Code Napoleon, in a suit instituted by a wife for divorce, on the ground that she had, immediately after marriage, contracted syphilis from her husband, *the suit was not successful.* The prosecutor cited the same articles of the code plead in the previous case, cited two cases sustained by the parliament of Metz; the views of La Prétre and Soesve. The court refused to grant a decree of separation. The court of Pau, February 3, 1806, to which it was appealed, declared the ground not to be one of the three causes allowed by article 134 of code—abuse, ill-treatment, grave injury. The Court of Cassation confirmed the judgments, declaring that the applicant for separation had not shown in evidence any circumstance that brought the case within the meaning of the law, *i. e.*, of what she complained: *excés, sevices, injures graves.* In this seems to lie the chief cause of contradiction with the courts: the law does not define what shall constitute ill-treatment or grave injury, or, as in the Ohio law, "extreme cruelty." The interpretation is left to the court. The circumstances only can then determine in the minds of the court the true nature of the case. What in one case would be a total disregard of all the duties of marriage—the innocence of the wife, her health, life, or character—with another case would be the contrary.

Thus, one knowing nothing of the nature of syphilis, might contract marriage in good faith, and communicate the disease, yet the case be stripped of all its worst features. We were consulted in a case of this kind a few years ago. A young man, who before marriage had been licentious, contracted a syphilis which became secondary. The appearances of the disease were suppressed. He consulted an old ignoramus, who declared it could be no obstacle to marriage. The young man had his misgivings; but on the advice of this *quasi* medical man, married. The result of the union was an idiotic, epileptic child, born to all appearances well developed, but soon becoming an object of pity, helpless. The wife became syphilitic, with all the appearances of secondary and tertiary syphilis. A servant girl who nursed the babe contracted syphilis from kissing it constantly; its lips being covered with syphilitic ulceration. The husband lay, at different periods, for months, helpless with syphilitic troubles, rheumatism, etc. Treatment would suppress, *but never eradicate* the disease.

A second case, laid before me a few days ago, has called forth this article. A minister of the gospel, in good standing, and of excellent moral character, applied to a lawyer in this State, where he resides, to conduct a suit for divorce, based on the grounds that he married a woman who *represented herself* to be virtuous, of fair character, etc.; but that she, before marriage, had been a prostitute, and in leading this public life, had contracted syphilis. I have been requested by the counsel for the husband to present an opinion upon the subject, based upon medico-legal writers. The counsel say that "The law books contain nothing of importance on the subject." This is true, if we consult American or English treatises for *direct opinions;* but if we seek for *general principles* to guide us, we will find sufficient. In French works, we find what the other writers fail to supply. The principal works consulted are those of Briande et Chaude, Fodere, and Devergie. We translate such portions as will throw light upon the case in question. Syphilis, under all circumstances and conditions, contracted from the brothel, is the just punishment of a parent's sin, and shows how clearly the sins of the parent may be visited, physically, mentally, morally, upon the offspring.

In the following views of the counsel, Linguet and the Advocate-General, Verges, submitted by the latter in 1771, in a case which explains itself, we find the main points presented that we desire: "What! for a passion that perhaps repentance has followed, a woman can escape from the empire of her husband, yet she can not after an outrage that causes a poison to circulate in her veins of which the remedies the most vaunted can not always destroy all the effects. Injurious epithets, pronounced in anger, have sufficed sometimes to deprive a husband of a wife, that in the depth of his heart he respects; but they treat carefully him who, without regard for the innocence of his wife, exposes her to the scandal and repulses of society. * * * *

"In truth, marriage is an association for good and for ill; but this association is not of the ills of which the source is libertinage, the origin of which is disgraceful. The diseases and infirmities which it pleases Providence to send, attack alike virtue and vice; their presence is announced by visible signs; attempted precaution and preservation can be successful. Syphilis, on the contrary, is the fruit and punishment of the debauchee. Here the contagion is concealed under the veil of affection. It is a crime for a wife to repel without motive the caresses of her husband,—is it not a crime for a husband to abuse the most sacred of ties? Why then, say we, so many judgments which have not admitted, or which have formally rejected this cause?

"*It is but necessary, in order to admit it, that the truth of facts be not problematic, that the origin of the evil be not doubtful, that its effects be not transient, or easily cured.* When a married couple accuse each other mutually, then an impenetrable confusion arises which conceals the source of infection; the judgment ought to be arrested, *not for insufficiency of cause*, but for that of proof. When, on the contrary, evidence is acquired that convincing facts manifests the truth, the separation is legitimate and necessary." The court decreed it. It was held at Pau, in 1806, that the law does "express, in precise and formal terms, the causes of separation. Venereal disease, not being expressed as one of the causes enumerated, *is thereby excluded*; that the causes ought not to be extended, but limited. The law having clearly defined the causes for separation, it is necessary to conclude that, from its silence concerning venereal disease, that it has not designed to make it a cause for separation: *quod tacuit, noluit.*"

Upon this defense, the court decided, February 6, 1807, "that the communication of venereal disease is not *essentially* a cause of separation of the bodies; *but at the same time, if the communication was accompanied with circumstances which gave it the character of ill-treatment or grave injury, it would be otherwise.*"

A judgment rendered in the Court of Lyons, April 4, 1816, was as follows: "Whereas, in itself and isolated from all particular circumstances, the communication of venereal disease ought not to be appreciated by tribunals as a grave injury, in the sense of the law, because the mest often it is involuntary—the husband not having a knowledge sufficient of his condition, and because it is often difficult to recognize what is the true origin of this clandestine and mysterious communication of his nature. But considering that, in the case all the circumstances present the character of injury the most grave for the dame V. of the outrage the most grievous for morals, and the most frightful for the family, since it comes to pass that a man knowingly infected with the disgraceful poison of the debauchee, has the infamy to defile the nuptial couch the very day he was admitted to it; of a man who has poured, with the full knowledge of the cause, the germ of this shameful disease into the bosom of the unfortunate her of whom he has deceived the faith; who has destroyed, from the first of her conjugal life, her physical and moral existence; who has thus borne in the heart and bosom of an entire family shame and despair, instead of the happiness he had promised; who has at length heaped up the measure of perversity in seeking to stifle the complaints and tears of his victim by acts the most serious. * * * The court confirms the first judgment, and allows to be admitted in evidence proof of the communication of the veneral disease."

Judgments might be cited similar to the above of the court of Besancon, February 1, 1808; Lyons, April 4, 1818; Toulouse, January 20, 1821; Paris, February 2, 1866; Paris, April 27, 1861. In this case the Advocate-General, Pinard, gave as opinion, which was sustained, that the communication of venereal disease is a grave injury and a cause of separation, and that it matters not whether the party communicating it contracted it before or after marriage, if there was knowledge of it and its contagious nature.—*Gaz des trib.*, 27 *Mai*, 1861.

Pothier (on Obligations) declares venereal disease not a cause of divorce (Marriage Contract, 515). Bishop (Marriage and Divorce, sec. 169) says: "A man takes a wife for 'better or for worse,' and waives all questions of wisdom, virtue and wealth. 'I take you to be my wife, whether you have the qualities or not, whether you have deceived me or not.' "

The law presumes that due caution has been used.

SEC. 179. "If a woman who has been defiled pretends to be a virgin, and a man marries her on his faith in this pretension, the marriage is nevertheless good, even though she be a common prostitute." For the reason that it is presumed that if she marries, she has abandoned her former vile associations, and intends to lead a virtuous life.

SEC. 180. Bishop says: "Antenuptial incontinence is no ground for divorce."

SEC. 697. The same writer says: "*Corporal infirmities which inspire disgust and repugnance, or which hinder the accomplishment of the ends of marriage, give to the other a right of divorce.*" What corporal infirmity is more loathsome, more disgusting than syphilis? What can hinder more, unless it be physical incapacity, the accomplishment of the ends of marriage? Shall a man be forced to cohabit with a female with no other hope than that the fruits of his union will be diseased, imperfect, idiotic, or other burdens upon him, who in their turn may affect others as innocent as themselves?

One or two points alone remain to be considered. In reference to evidence: How shall the existence of venereal disease be proved? If the husband alleges the wife to be infected, can she be compelled to undergo an examination of experts? We know of no law to compel her to expose her person to examination; nor do we know that a refusal to do so can justly be presumed to be evidence against her.

Again the existence of venereal disease being proved beyond a doubt, contracted after marriage, to what extent shall it be admitted as evidence of *adultery?* Such has been held to be a legitimate deduction in French cases. The allegation of "extreme cruelty" requires some explanation. Trielhard, who has annotated the French Code, interprets this to mean, not temporary freaks of passion, but such ill-treatment as would render life insupportable. By the Ohio reports, *words alone* can not constitute "extreme cruelty" (Wright, 212), but personal violence must be inflicted. Courts have declared that the degree of abuse necessary to constitute ill-treatment sufficient to be a cause of divorce varies with the condition in life and social relation of the parties; thus those in the lower walks of life often submit daily, without complaint, to ill-treatment that others, in a higher social position, would not tolerate for a single day. There are thousands content to drag out an existence, feeling none of those higher sentiments that spring from a well-constituted moral nature. Ignorant of the existence of a higher life, their ignorance to them is bliss. Excesses, ill-treatment, extreme cruelty, are words unknown, or, if known, incomprehensible; for their measure of life is far short of the refined measure of more civilized life.

In this grade of life it is rare that courts are called upon to adjust matrimonial difficulties. We see daily in our practice, even here in the country, a want of harmony, a failure to comprehend the true nature and relation of married life, that if existing in the higher circles of a metropolis, would soon terminate the "contract" to live for "better or for worse" as companions. But what would be the result with this class, if a marriage was set aside? Simply that both parties, perhaps, would contract a worse one, and other questions be raised.

In mining districts, in the suburbs of cities, with tenants of foreign extraction, often marriage, instead of being the basis of union, is a perpetual disunion, discord, strife; instead of home, the abode is little less than a conjugal hell. Words, then, should not constitute grounds for divorce; but the "extreme cruelty" should be more forcible measures, for in divorce suits it is personal violence. (Wright, 563.)

It is not the object of this article to discuss the sixth cause of divorce, given in the statutes: "Fraudulent contract." The cases cited, or points in question, might properly be considered in this light. In marriage, the law presumes that both parties are capable of executing the duties implied by the marriage contract, for the duties are not expressed, but implied. In case a loathsome disease renders the accomplishment of these duties impossible, then the party aggrieved has a claim for redress of the wrongs inflicted. A party knowing himself inflicted with syphilis, its contagious character, the injury it produces, could not perpetuate a more outrageous fraud upon an innocent woman than to marry her, concealing this knowledge from her.

Neither do we desire to discuss the subject under the light of an "error," a question of *personal identity*. Thus, if a man marries one woman, supposing he has married another, there is "error." Marrying one *impotent* has been held to be an error. In the absence of statutory enactments, a suit can be brought in several different ways that legal enactment may prevent. In Ohio, the statutes declare impotency a cause of divorce; hence actions under the common law can not be maintained.

In conclusion, what shall we declare to be the result of our research? Is venereal disease, contracted before marriage, a cause of divorce? Will it render such marriage *void?* No; nor under all circumstances *voidable*. That is *void* that never had legal value; null *ab initio*. A *voidable* marriage is good until it has been set aside. After marriage, if syphilis is contracted, it involves other questions, as *adultery*, etc. Under most circumstances, we believe that a marriage contracted without the knowledge of the existence of venereal disease, would entitle the party deceived to a judgment of divorce. We see no good reason why any court should refuse to decree it. But, on the other hand, if a party fails to use due caution, marries one known to be a prostitute, and contracts syphilis thereby, no court should relieve him of his burden. It would not be *voidable.* Want of due caution would render it neither an "error" nor a "fraud."

Many other questions might be raised and points presented, but we shall not extend the paper further. If the second case presented be prosecuted to a final judgment, we will present the main points raised, in this journal, as well as the rulings upon points of evidence that *must of necessity* call for judicial opinion. Hoping the matter presented may interest all who are interested in medico-legal research, we submit all further consideration to the individual opinion and observation of the reader.—*Cincinnati Lancet and Observer.*

